# Venous Thromboembolism in a Young Girl with Duplication of the Inferior Vena Cava and Protein S Deficiency

**DOI:** 10.4274/tjh.galenos.2019.2018.0332

**Published:** 2019-05-03

**Authors:** Wei-Li Liao, Ming-Yang Shih, Jiaan-Der Wang

**Affiliations:** 1 Department of Pediatrics, Taichung Veterans General Hospital, Taichung, Taiwan; 2 Center for Rare Disease and Hemophilia, Taichung Veterans General Hospital, Taichung, Taiwan; 3 Tunghai University, Faculty of Medicine, Department of Pediatrics, Taichung, Taiwan

**Keywords:** Vena cava, Protein S deficiency, Venous thromboembolism

## To the Editor,

**Figure 1:**
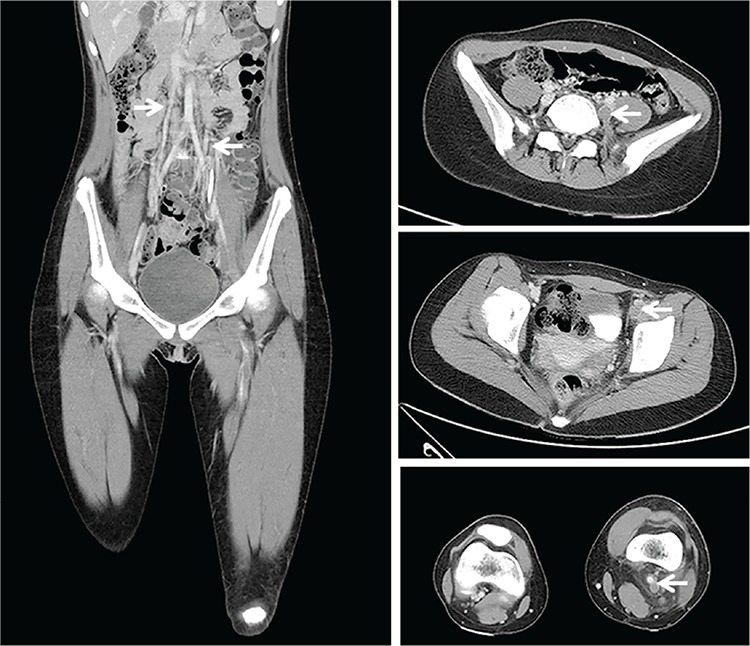
Left panel: Contrast-enhanced computed tomography image demonstrating duplicated inferior vena cava (whitish arrow). Right panel, from top to bottom: Arrow indicates thrombosis found in engorged left iliac vein, femoral vein, and popliteal vein. 254x190 mm (72x72 DPI).

A previously healthy 13-year-old girl presented with a 3-day history of progressive swelling and pain in her left lower limb. She also complained of cough in the last 2 weeks. No trauma, surgery, travel, or medication was noted before this illness. Physical examination revealed significant swelling and tenderness in her left lower limb. The laboratory data showed a high level of D-dimer (13.0 mg/L FEU, reference range <0.55 mg/L FEU). Multidetector computed tomography showed extensive emboli formation from the left calf region to the left ilio-femoral veins and duplication of the inferior vena cava (IVC) (Figure 1). Pulmonary ventilation-perfusion (V/Q) scintigraphy revealed several mismatched areas diagnostic for bilateral acute pulmonary embolism. Tracing the family history, her father had developed venous thromboembolism (VTE) at the age of 40 years and was diagnosed with protein S deficiency. A thrombophilia screening in this patient identified severe protein S deficiency (protein S activity: 2%, reference range: 55%-140%). Other results, including levels of homocysteine, antithrombin III, and protein C activity, were within normal limits; factor II G20210, factor V Leiden G1691A, anti-cardiolipin antibody, and anti-β2-glycoprotein I IgM and IgG were all negative. Her symptoms and signs subsided after treatment with heparin, followed by warfarin for 3 months. The repeated measurement of protein S activity was 7% after discontinuation of treatment with warfarin for one week. Given that two provoking risk factors were present, the patient continued to receive prophylactic therapy with warfarin.

Virchow’s triad describes the three main factors contributing to thrombosis, which include hypercoagulability, vessel injury, and venous stasis. Congenital anomalies of IVC may predispose to VTE due to resultant venous stasis. Duplication of IVC is usually considered as asymptomatic and an incidental finding while performing retroperitoneal surgery or venous interventional radiology. However, an increasing number of studies suggest that cases of unprovoked VTE were associated with duplication of the IVC [1,2,3,4]. The ages of these patients ranged from 18 to 84 years. No pediatric patient was reported. 

VTE is long considered to be far less common in children than in adults. Most pediatric VTE is provoked and occurs with multiple risk factors [5]. Genetic risk factors play an important role in children who develop VTE and thrombophilia screening is suggested in selected patients with VTE, such as young patients [6]. Protein S deficiency leads to loss of control of thrombin generation and fibrinolysis, and is associated with 5.8-fold increased odds of index VTE [7]. VTE in unusual sites has unique and obscure provoking factors [8]. Therefore, protein S deficiency was an important risk factor for the VTE event in this patient. In summary, we hypothesize that duplication of the IVC and protein S deficiency both promoted intravenous thrombus formation and predisposed the patient to develop VTE at a younger age. The combination of a rare congenital thrombophilic trait with a rare anatomic variant is very infrequent. In young patients with VTE less common causes of thrombosis such as inherited thrombophilias and anatomic abnormalities should be considered.

## References

[1] Anne N, Pallapothu R, Holmes R, Johnson MD (2005). Inferior vena cava duplication and deep venous thrombosis: case report and review of literature. Ann Vasc Surg.

[2] Milani C, Constantinou M, Berz D, Butera JN, Colvin GA (2008). Left sided inferior vena cava duplication and venous thromboembolism: case report and review of literature. J Hematol Oncol.

[3] Saad K, Saad P, Amorim CA, Armstrong D, de Freitas Soares BL, Neves PCF, Filho AR (2012). Duplication of the inferior vena cava: case report and a literature review of anatomical variation. J Morphol Sci.

[4] Lambert M, Marboeuf P, Midulla M, Trillot N, Beregi JP, Mounier- Vehier C, Hatron PY, Jude B (2010). Inferior vena cava agenesis and deep vein thrombosis: 10 patients and review of the literature. Vasc Med.

[5] Van Ommen CH, Heijboer H, Büller HR, Hirasing RA, Heijmans HS, Peters M (2001). Venous thromboembolism in childhood: a prospective two-year registry in the Netherlands. J Pediatr.

[6] Colucci G, Tsakiris DA (2017). Thrombophilia screening: universal, selected, or neither?. Clin Appl Thromb Hemost.

[7] Young G, Albisetti M, Bonduel M, Brandao L, Chan A, Friedrichs F, Goldenberg NA, Grabowski E, Heller C, Journeycake J, Kenet G, Krümpel A, Kurnik K, Lubetsky A, Male C, Manco-Johnson M, Mathew P, Monagle P, van Ommen H, Simioni P, Svirin P, Tormene D, Nowak-Göttl U (2008). Impact of inherited thrombophilia on venous thromboembolism in children: a systematic review and meta-analysis of observational studies. Circulation.

[8] Shatzel JJ, O’Donnell M, Olson SR, Kearney MR, Daughety MM, Hum J, Nguyen KP, DeLoughery TG (2019). Venous thrombosis in unusual sites: A practical review for the hematologist. Eur J Haematol.

